# An Evaluation of Mental Health First Aid Officer Utilisation Across an International, Interdisciplinary Oncology Group

**DOI:** 10.1002/jmrs.70021

**Published:** 2025-09-09

**Authors:** Janeane Summerfield, Aidan Leong

**Affiliations:** ^1^ Bowen Icon Cancer Centre Wellington New Zealand; ^2^ Icon Group Brisbane Australia; ^3^ University of Otago Wellington New Zealand

## Abstract

**Introduction:**

Mental health is a critical component of overall well‐being, yet healthcare professionals, particularly those in oncology, face unique stressors that increase their risk of compassion fatigue and burnout. Mental Health First Aid (MHFA) training equips non‐mental health professionals with the skills to support individuals experiencing distress. Our international oncology organisation implemented MHFA training to foster mentally healthy workplaces. This study evaluated the utilisation of these MHFA officers (MHFAOs) over a 12‐month period.

**Methods:**

An anonymous online survey was conducted in July 2024, inviting 34 trained MHFAOs across 13 professional disciplines. The survey collected quantitative and qualitative data on MHFA training, utilisation and perceptions.

**Results:**

A total of 21 survey responses (62%) were included for analysis. While most respondents had engaged in MHFA interactions, six reported no interactions in the past year. The majority of MHFAO reported the combined interactions totalled between 3 and 5 h, with some exceeding 10 h total in the past 12 months. While most respondents reported manageable MHFA workloads, key challenges identified in the qualitative data include a potential lack of awareness of the MHFA programme, unclear role definitions and responsibilities, and limited resources.

**Conclusions:**

Utilisation of MHFA across Icon's international, interdisciplinary oncology service in Australia and New Zealand has been demonstrated. While most respondents reported manageable MHFA workloads, it was identified that challenges exist in programme awareness, role clarity, and resource allocation. Addressing these concerns through improved guidelines, ongoing training, and increased visibility of MHFAOs could enhance the programme's effectiveness and long‐term sustainability.

## Graphical abstract

Mental Health First Aid in an oncology setting – a survey of utilisation over a 12 month period.

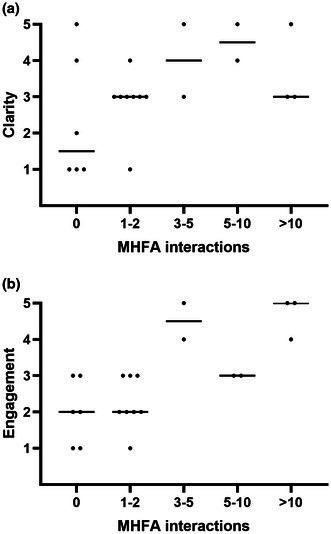

## 1 Introduction

Good mental health is a key component of overall health and well‐being for people, meaning better ability to connect, function, cope and thrive [1]. Oncology health professionals endure some of the most emotionally demanding healthcare environments with unique stressors including exposure to patient suffering and providing end‐of‐life care [2]. These challenges can lead to mental health deterioration such as emotional exhaustion, compassion fatigue, and burnout especially when combined with high patient load and understaffing [2]. Mental health challenges affect healthcare workers' well‐being, patient care quality, and service delivery due to staff absenteeism or turnover, making workplace mental health a global priority in healthcare [2, 3]. Mental health challenges are widespread, with 41% of New Zealand (NZ) adults in the 2024 NZ health survey reporting seeking support for concerns regarding their mental health and/or problematic addictions [4]. The Health and Disability Commissioner 2018 report states that within a year, one in five NZ adults will meet the diagnostic criteria for a mental health and/or addiction condition [5]. Early mental health intervention improves outcomes for those experiencing distress, and with support, people often recover from mental health challenges and regain well‐being [6]. Health professionals endure some of the most emotionally demanding healthcare environments with unique stressors including exposure to patient suffering and providing end‐of‐life care [2]. These challenges can lead to mental health deterioration such as emotional exhaustion, compassion fatigue and burnout especially when combined with high patient load and understaffing [2].

Given the high prevalence of mental health disorders and barriers that can prevent individuals from seeking help from a professional; individuals in a social network may be ideally placed to identify or initiate conversations about mental health changes in each other [7]. Work colleagues fit this description well because full‐time employees spend up to 40% of their waking hours at the workplace, integrating socially with their colleagues and building social networks [8]. Training non‐mental health professionals in Mental Health First Aid (MHFA) began in Australia in 2000 and in New Zealand since 2010. Similar in concept to physical first aid, MHFA officers deliver initial support in the workplace to help those experiencing mental health challenges and to help in a mental health crisis until professional mental health help is received [7, 9]. This training improves mental health awareness, knowledge and confidence to help amongst participants. It is also designed to reduce stigmatising attitudes, social distancing and increase mental first aid actions towards those experiencing mental health challenges [6, 10, 11]. Of note, a recently published Cochrane review on MHFA as a tool for improving mental health and well‐being, the authors were unable to draw conclusions on the effects of MHFA training due to a lack of quality evidence [11].

Icon Group (Icon) brings together all aspects of quality cancer care, including medical oncology, radiation oncology, haematology, pharmacy and chemotherapy compounding to deliver cancer services for cancer patients around the world. Mental Health First Aid officer (MHFAO) roles at Icon were established in Australia (2020) and New Zealand (2023) as part of the Icon strategy for developing mentally healthy workplaces. The Mental Health First Aid programme is just one resource that falls under the umbrella of the Icon workplace wellbeing programme. Any staff member experiencing mental health distress is encouraged to contact the internally listed trained MHFAO for support (or with external counselling services). A list of all the trained MHFAOs is available on the intranet by name, location, role, contact details, and division/department. Employees facing mental health challenges are encouraged to reach out in person or online, depending on time zone. The MHFAO roles span 13 different clinical and non‐clinical disciplines across the compounding, pharmacy, cancer centres, and head office divisions. The aim of this study was to evaluate the utilisation of MHFAOs at Icon over a 12‐month period.

## 2 Methods

An anonymous survey was created using Microsoft Forms to develop and administer a mixture of closed and free‐text response survey questions to gather respondents feedback and insights on MHFA training, utilisation, and perceptions (Appendix S1). Survey participants were aware through the participant information sheet that submission of the survey confirmed consent and that participation was voluntary. Ten multichoice questions assessed training, interactions, and perceptions of the MHFA programme. Three free‐text questions were asked regarding what works well, challenges faced, and any other feedback or suggestions on respondents' experience of utilising their MHFA skills in the workplace. The online survey was administered via Microsoft Forms over a 3‐week period within July 2024, with 2 reminder emails to invited participants during the timeframe. Thirty‐four MHFAO Icon employees, across 13 professional disciplines, were invited to participate. They were all selected and invited from a central database published on the Icon intranet. One author is an MHFAO and was excluded from participation in the survey. Participation was voluntary and anonymous. Ethical approval was granted by the University of Otago Human Ethics Committee (reference 24/0280).

### 2.1 Data Analysis

Survey responses were analysed using Microsoft Excel. Descriptive statistics were calculated for multichoice questions by the authors to analyse quantitative data collected from the survey. The free‐text comments were analysed using directed content analysis [12] and were initially coded and organised into a summary table. A second and third coding cycle compiled statements into themed categories. Three clear information categories were then identified for reporting the results. The final coding cycle brought awareness of anchor statements that thoughtfully exemplified these main three categories for publication. A review of the themes was performed to ensure logical alignment with the original survey context by the authors independently and collaboratively. One researcher initially performed the coding and this was independently reviewed by the second researcher to ensure trustworthiness. Both authors met to discuss and coordinate their analysis and results. Discussions on differing interpretations and ambiguity were had until consensus was reached. One researcher was involved in the Icon MHFAO programme which gave valuable insight into the interpretation of the data. However, it is acknowledged this inherently influences the perspective brought to the interpretation of the qualitative data. The second researcher who reviewed the coding was not involved in the Icon MHFA programme.

## 3 Results

Twenty two survey responses were received. One response was from an unintended recipient outside the MHFA programme and was censored, resulting in 21 responses available for analysis from 34 invited participants (62% response rate). Respondents reported their formal MHFA training took place predominantly between 2020 and 2023, and two MHFAO had completed refresher training (Table 1).

**TABLE 1:** Completion of MHFA training.

	*N*	%
Year of MHFA training		
2024	1	5%
2023	5	24%
2022	9	43%
2021	3	14%
2020	3	14%
Refresher MHFA training received		
Yes	2	10%
No	19	90%
Total	21	100%

Respondents reported the number and total time of MHFA interactions with staff in the past 12 months (Table 2). Six respondents (29%) reported zero interactions. The most frequent response (38%) was 1–2 interactions over the last 12‐months, though three staff (14%) did report more than 10 interactions. In regards to the time spent on MHFA interactions, six respondents (32%) reported 3–5 h over the last 12‐months. A further four respondents (19%) reported spending more than 5 h on MHFA interactions, with three of these spending more than 10 h.

**TABLE 2:** MHFA interactions over a 12‐month period.

	*N*	%
Number of MHFA interactions		
0	6	29
1–2	8	38
3–5	2	10
6–10	2	10
> 10	3	14
Time spent on MHFA interactions		
N/A	6	29
< 1 h	2	10
1–2 h	2	10
3–5 h	7	33
5–10 h	1	5
> 10 h	3	14

Abbreviation: MHFA, mental health first aid.

As shown in Table 3, of the 15 respondents that had engaged in MHFA interactions, the majority (67%) had supported escalation to external referrals (e.g., for professional help). While 1–2 interactions requiring escalation was most frequently reported (53%), one respondent (7%) reported they had facilitated over ten external referrals. Thirteen respondents (87%) indicated that the primary theme of MHFA interactions with staff were a combination of non‐work and work‐related issues. All respondents (100%) reported that either, ‘most’ or ‘all’ MHFA interactions were with staff already known to them. 60% of respondents reported that ‘most’ or ‘all’ of MHFA interactions were initiated by staff seeking support, as opposed to them initiating interactions as a MHFAO.

**TABLE 3:** Characteristics of MHFA interactions over 12‐month period.

	*N*	%
Primary theme of MHFA interactions		
Work‐related	0	0%
Non‐work‐related	2	13%
Combination	13	87%
MHFA interactions requiring escalation		
0	5	33%
1–2	8	53%
3–5	1	7%
5–10	0	0%
> 10	1	7%
Prior familiarity to staff within MHFA interactions		
All interactions with known staff	11	73%
Most interactions with known staff	4	27%
Most interactions with unknown staff	0	0%
All interactions with unknown staff	0	0%
Initiator of MHFA interactions		
All initiated by staff seeking support	1	7%
Most initiated by staff seeking support	8	53%
Most initiated by MHFAO	4	27%
All initiated by MHFAO	2	13%

**FIGURE 1:**
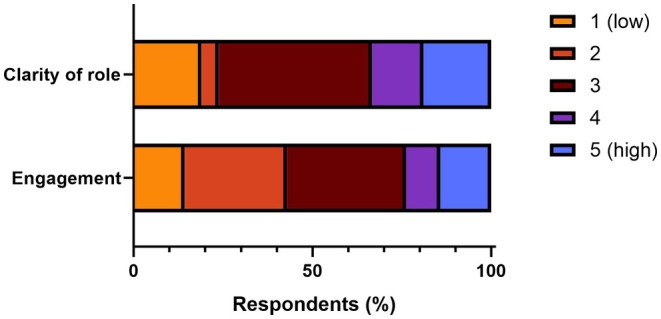
Perceptions of MHFAO role clarity and programme engagement.

Respondents were asked to rate the clarity of their role as well as engagement with the broader MHFA programme (Figure 1). Five respondents (24%) reported low [1, 2] clarity of role, with the largest proportion (43%) reporting moderate clarity. Nine respondents (43%) reported low [1, 2] engagement with the MHFA programme, with a further 33% reporting moderate engagement.

**FIGURE 2:**
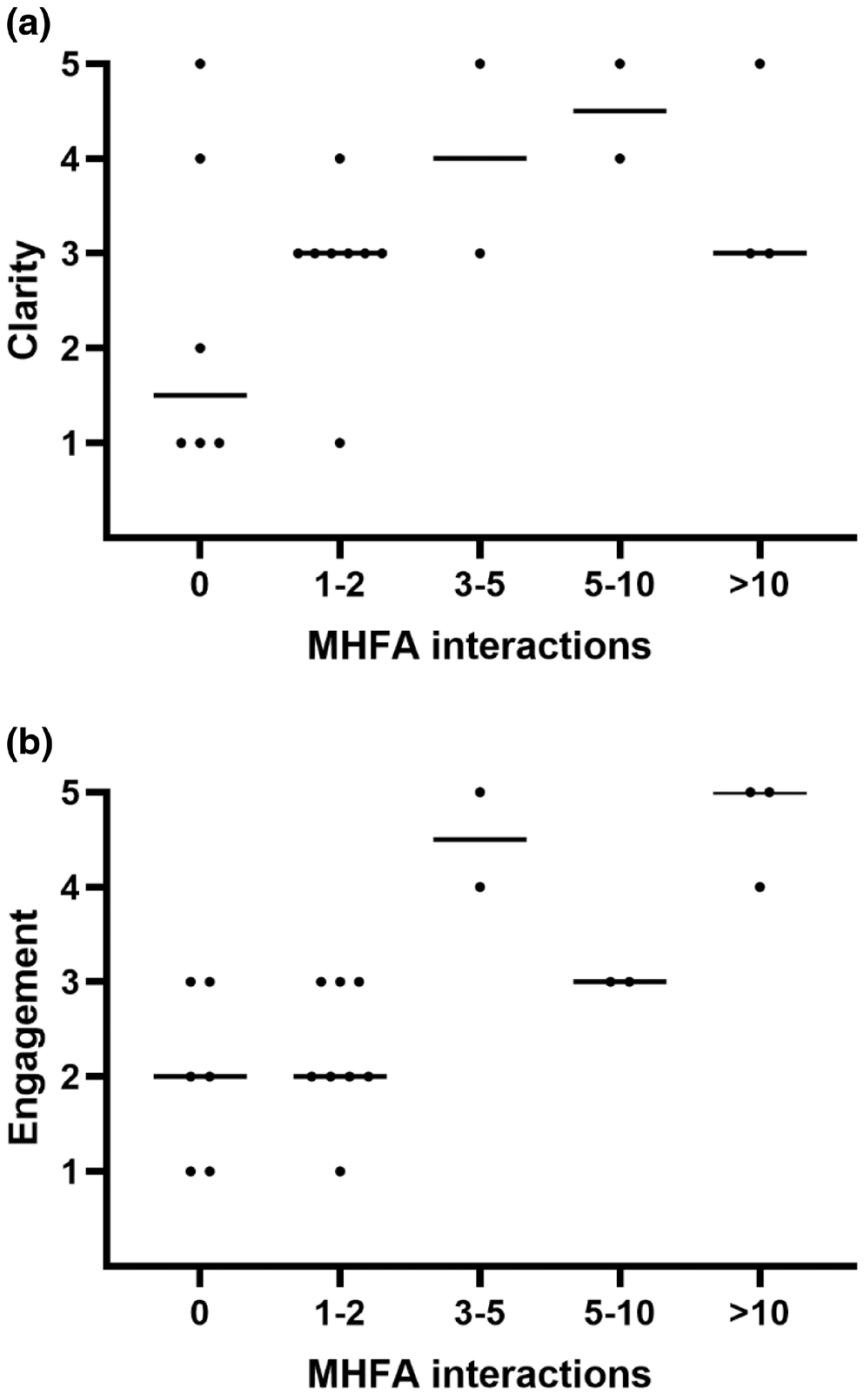
Association between MHFAO perceptions and volume of MHFA interactions. Respondents perceptions of role clarity (a) and programme engagement (b). Each dot represents an individual respondent, solid lines indicating the median value of a column.

Figure 2 shows responses to clarity of role and engagement plotted against reported number of MHFA interactions. While a range of role clarity was shown across respondents' volume of MHFA interactions, engagement with the MHFA programme was seen to be lowest amongst those who reported 0 to 2 interactions, and highest amongst those reporting > 10 interactions.

Three key themes were identified through coding of free‐text questions using directed content analysis [12]. These were resourcing, awareness and scope.

The first theme identified was ‘resourcing’. Respondents highlighted challenges with time, location, tools and resources for helping colleagues in distress. Respondents also raised a desire for ongoing financial assistance for MHFA refresher training.

[a challenge faced with the MHFA role is] setting aside time during work hours to speak and interact with individuals who require assistance. Respondent 19

The challenges I face is knowing what organisations to reach out to or recommend for mental health services. If there could be a resource that we can refer to when trying to help a person who requires this service that might be helpful. Respondent 1

Having private space for comfortable conversations, offices feel instense {sic} and our site isolation means nowhere to go ‘for a coffee’ to try and facilitate casual conversations. Respondent 11

Secondly, ‘awareness’ was an emergent theme relating to concerns regarding the lack awareness of the MHFA programme across teams.

… I think the awareness isn't there for many people. I could probably get my certificate out or pop a sticker up, email signature etc. to get more awareness out there. I also haven't managed to get to meetings due to conflicting schedules. Respondent 15

As a MHFA I send out emails that hopefully lets people know that I am here if needed. Respondent 8

People don't really understand what it means. Respondent 22

Thirdly, ‘scope’ was defined regarding clarity of roles and responsibilities for those respondents trained in MHFA. Some survey respondents reported value in using their skills with both colleagues and patients alike.

This ties in well with my duties as nurse manager, with goal setting and individual counselling of staff and patients. Respondent 12

We use this for patients as well as staff. Respondent 13

## 4 Discussion

This study has shown a range of respondents' experiences by interdisciplinary staff trained as MHFAO across an international oncology organisation. While a limited number of responses were collected from a small cohort, which limits the ability to draw robust conclusions supported by statistical analysis, the findings are nonetheless valuable to inform the value of the programme as well as opportunities for future development. The survey utilised was intentionally designed as a short‐format evaluation, which inevitably reduced the depth of data that could be collected from respondents. A more comprehensive survey would enable additional detail to be explored regarding the role of the MHFAO, particularly regarding opportunities to promote engagement with MHFA services and how to best support and sustain MHFA practitioners themselves. Qualitative approaches such as semi‐structured interviews would likely add significant value in understanding the MHFAO experience. Respondents' MHFA interactions that took place during the survey period were predominately reported to have been with people already known to the trained MHFAO. The vast majority (87%, excluding those who had no interactions) said their MHFA interactions were a combination of personal and work‐related issues. This result aligns with the 2022 World Health Organisation report where it states that a shift (positive or negative) for an individual's mental health is linked to a diverse range of individual, family, community and structural factors [1]. Work‐home conflict exists in various degrees for individual healthcare workers with both organisational and personal factors associated with work engagement [2, 13].

Survey respondents' perceptions of the MHFA programme at Icon showed a spread of responses regarding the clarity of their roles and responsibilities and a trend of overall lower engagement with the Icon MHFA programme, particularly for those with two or fewer interactions over the last 12 months. While it is understandable that a lower use of MHFA skills would diminish engagement in the programme, promotions to increase awareness and utilisation of MHFAOs within Icon Group may have the dual benefit of increasing both clarity of role and engagement with the programme for MHFAOs—strengthening the MHFA programme's impact overall.

Interestingly, one respondent in the survey also identified challenges as a MHFAO that also was in a management role. The respondent highlighted the challenges of individuals with leadership responsibilities when needing to engage in managing the ongoing support response to the mental health challenges disclosed by the team member. However, there is potential for conflict if the same manager may have been privy to any disclosed mental health challenges with the member of their team within their scope as a trained MHFAO person. Although this is a sub‐theme in this survey, the wider literature indicates similar challenges are experienced in a range of other organisations [14]. This includes challenging experiences of line managers in maintaining a sense of professional distance while also helping their employee deal with their mental health issue. Also reported in the literature was the challenge of cross‐pressure in managing the needs of the employee (protecting privacy) with the needs of other workplace colleagues (who may have increased workloads as they cover their absences and decreased job performance). Additional cross‐pressure occurs for line managers to balance the commercial and operational demands of the organisation with the needs of the employee experiencing mental health challenges [14]. The literature further highlighted the critical need for organisational support and clear guidance about the appropriate steps to take for line managers so that effective support processes unfold for employees with mental health challenges [14].

It can be inherently difficult to define the roles and responsibilities for MHFAO that hold clinical roles. This is because some MHFA survey respondents were inadvertently identified as clinical staff when they reported using their MHFA skills with their distressed oncology patients, as well as colleagues. This is a logical use of MHFA skills, and clinical staff are skilled at delivering patient care with a holistic approach. However, this is potentially outside of the current scope of MHFA training boundaries, where the disclaimer of MHFA training states it should not be a substitute for professional mental health advice [9]. Oncology patients may perceive mental health support as professional advice because they are in a healthcare environment for their cancer treatment. Clinical staff need to communicate boundaries clearly with patients in such situations. Similar issues are also identified in the wider MHFA literature, where the solution is adapted MHFA programmes for various contexts, including frontline healthcare professionals, with the efficacy of these adapted programmes still being evaluated [15, 16, 17]. Within Icon, further studies are indicated to explore if the current MHFA programme is aiding the Icon group goal of fostering mentally healthy workplaces.

## 5 Application to Practice

These survey results gained valuable direct information from study participants and inform future development of the MHFA programme and a mentally healthy workforce at Icon group.

Suggestions for application to practise and future work are listed below:

1. Programme awareness: MHFAO championing mental health within departments and being given resources to lead workplace activities and initiatives.
2. Role clarity: Develop guidelines with specific detail for those with dual MHFAO and senior leadership roles so the appropriate steps are followed for effective support processes.
3. Resource allocation: Improve resources for tailoring referral recommendations such as a centralised referral database, schedule regular protected time for MHFAO, and create private spaces for conducting MHFA interactions.
4. Improving MHFAO communication: To boost engagement with the programme, communication is key to MHFAO sharing experiences.
5. Ongoing Training: mandatory refresher courses to keep MHFAO certified and equipped with up‐to‐date skills.

Overall, our study has demonstrated utilisation of a MHFA team towards supporting their Icon colleagues experiencing mental health challenges. The interdisciplinary approach currently includes those in clinical and non‐clinical roles, from management level to junior staff. This diversity reduces the resource conflict that can be experienced if a clinical staff member is unable to step away from their daily role to assist a colleague experiencing MHFA distress. Although the survey results showed variable clarity, programme engagement, and interactions, more than two‐thirds of staff had utilised their training to support a colleague, invested time in the provision of this support, and many were able to escalate to additional support services.

## 6 Conclusion

This study has demonstrated utilisation of Mental Health First Aid (MHFA) across Icon's international, interdisciplinary oncology service in Australia and New Zealand. While most respondents reported manageable MHFA workloads, it was identified that challenges exist in programme awareness, role clarity, and resource allocation. Addressing these concerns through improved guidelines, ongoing training, and increased visibility of MHFAOs could enhance the programme's effectiveness and long‐term sustainability. Future research should explore the programme's effectiveness from the perspective of the wider Icon workforce and investigate the long‐term outcomes of MHFA interventions on oncology professionals' well‐being.

## Ethics Statement

Ethical approval was granted by the University of Otago Human Ethics Committee (reference 24/0280).

## Conflicts of Interest

The authors declare no conflicts of interest.

## Supporting information


**Data S1:** Copy of survey questions.

## Data Availability

Data available on request from the authors.
