# Supplementary material for: An Evaluation of Mental Health First Aid Officer Utilisation Across an International, Interdisciplinary Oncology Group
Source: J Med Radiat Sci. 2025 Sep 9;72(4):450–5. doi: 10.1002/jmrs.70021 (PMC12661068; doi:10.1002/jmrs.70021)
Supplement: Supplementary file 1 — Data S1: Copy of survey questions. [file JMRS-72-450-s001.pdf]

How is Mental Health First Aid (MHFA) training being utilised at ICON Group?

Thank you for showing an interest in this project. Please read the provided participant information sheet carefully before deciding whether or not to participate. If you decide to participate we thank you. If you decide not to take part there will be no disadvantage to you and we thank you for considering our request.

What is the Aim of the Project?

The aim of this survey is to benchmark Mental Health First Aid (MHFA) practice at ICON group by staff currently trained in MHFA.

1. What year did you complete your MHFA training for ICON? *

- ○ 2020
- ○ 2021
- ○ 2022
- ○ 2023
- ○ 2024
- ○ Other

2. Have you completed any refresher MHFA training? *

- ○ Yes
- ○ No

3. Over the last 12-months, how many MHFA interactions with ICON staff have you supported? *

For the purposes of the survey, multiple conversations with an individual about the same issue is defined as one interaction. Subsequent conversations with an individual regarding different issues are defined as separate interactions.

- ○ 0
- ○ 1-2
- ○ 3-5
- ○ 5-10
- ○ More than 10

4. Over the last 12-months, approximately how long have you spent in total supporting ICON staff through MHFA interactions *

- ○ Less than 1 hour
- ○ 1-2 hours
- ○ 3-5 hours
- ○ 5-10 hours
- ○ More than 10 hours

5. Over the last 12-months, approximately how long have you spent in total supporting ICON staff through MHFA interactions *

- ◯ Less than 1 hour
- ◯ 1-2 hours
- ◯ 3-5 hours
- ◯ 5-10 hours
- ◯ More than 10 hours

6. Over the last 12-months, approximately what proportion of your MHFA interactions involved supporting individuals that were already known to you as a colleague? *

- ◯ All MHFA interactions with individuals already known to me
- ◯ Most MHFA interactions with individuals already known to me
- ◯ Most MHFA interactions with individuals previously unknown to me
- ◯ All MHFA interactions with individuals previously unknown to me

7. Over the last 12-months, approximately what proportion of your MHFA interactions involved an individual initiating contact with you to discuss support (as opposed to you initiating contact with them to discuss support)? *

- ◯ All MHFA interactions initiated by the individual
- ◯ Most MHFA interactions initiated by the individual
- ◯ Most MHFA interactions initiated by me
- ◯ All MHFA interactions initiated by me

8. Over the last 12-months, please indicate the number of times you needed to facilitate additional advice or referral outside of the workplace in relation to a MHFA interaction *

- ◯ 0
- ◯ 1-2
- ◯ 3-5
- ◯ 5-10
- ◯ More than 10

9. Of the MHFA interactions you have supported over the past 12 months, please indicate whether the issues raised were primarily work-related, non-work-related, or a combination of both. *

- ◯ Work-related
- ◯ Non-work-related
- ◯ A combination of both

10. As a trained MHFA person at ICON, please rate how clearly defined you feel that your role and responsibilities are *

1	2	3	4	5

Not well-defined at all — Very well-defined

11. Please comment on your experience of what works well with your MHFA role within ICON *

Enter your answer

12. Please comment on any challenges you have faced with your MHFA role within ICON *

Ensure that no identifying information is provided in relation to MHFA interactions

Enter your answer

13. Please rate your overall engagement with the ICON MHFA programme *

1	2	3	4	5

Not engaged at all — Very engaged

14. Any further feedback or suggestions are welcome (optional)

Enter your answer
